# More than meets the eye: Not a cardiac myxoma

**DOI:** 10.1016/j.xjtc.2025.06.022

**Published:** 2025-07-03

**Authors:** Akshat Saxena, Michael Seco, Charles G. Jenkinson, Peter W. Grant

**Affiliations:** a Department of Cardiothoracic Surgery, Prince of Wales Hospital, Randwick, Australia; b Discipline of Surgery, University of New South Wales, Kensington, Australia

**Figure:**
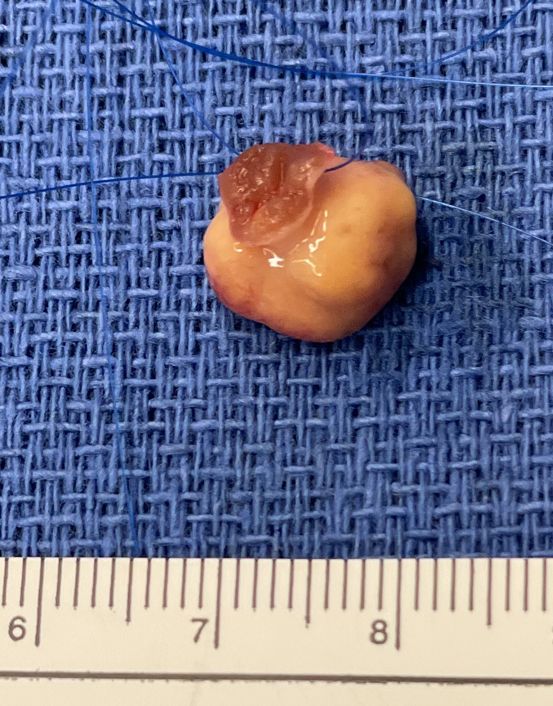
Resected left ventricular mass with scale.

**Central Message**

An index of suspicion is required for patients with a very distant history of renal cell carcinoma who present with an intracardiac mass.

## Clinical Summary

**Figure 1:**
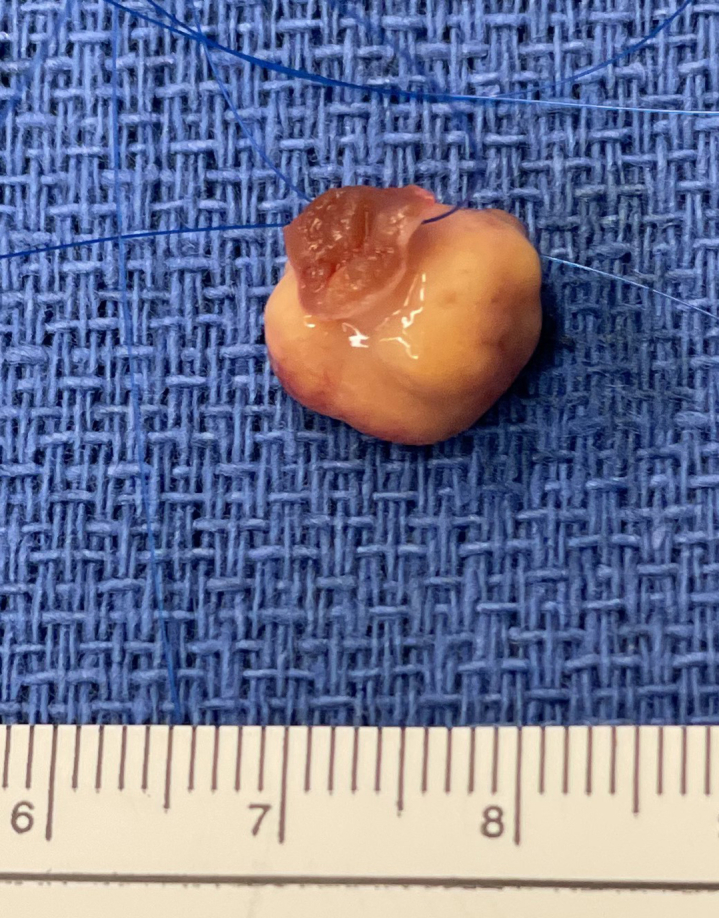
Resected left ventricular mass with scale.

A 67-year-old asymptomatic woman presented for an elective transthoracic echocardiogram (TTE). She had a distant history of right breast lumpectomy with radiotherapy 18 years ago for breast carcinoma and left nephrectomy for a renal cell carcinoma (RCC) 19 years ago. She underwent yearly breast mammogram/ultrasound by an oncologist and was deemed clear of any recurrent disease. The remaining right kidney was normal. The TTE demonstrated normal biventricular function with no valvular disease. However, there was a hypermobile left ventricular (LV) mass measuring 12 × 10 mm attached to the interventricular septum. On detailed review, the previous TTE showed a smaller mass in the same location 2 years ago. A transoesophageal echocardiogram (Video 1) confirmed a smooth, irregular mass attached by a stalk to the proximal anterior septum and in diastolic contact with the anterior mitral valve leaflet. A coronary angiogram excluded obstructive coronary artery disease but showed that the LV mass demonstrated contrast blush (Video 2). A cardiac multidisciplinary meeting established the working diagnosis as myxoma with differential diagnoses of papillary fibroelastoma, thrombus, and metastasis less likely. Given the high risk of embolization, obstruction, and valve dysfunction the consensus was for surgical extirpation. The procedure was performed via sternotomy and standard cardiopulmonary bypass with cardioplegic diastolic cardiac arrest. No pericardial effusion consistent with a malignancy was observed. The tumor was exposed via an oblique aortotomy and excellent visualization of the mass was obtained by retracting the right coronary cusp of the aortic valve. A 5-0 nylon suture was placed through the base of the stalk of the tumor. Using a 15 blade and scissors, a small myectomy was performed at the base of the tumor allowing complete excision (Figure 1). After closure of the aortotomy, the crossclamp was removed and the patient was weaned off cardiopulmonary bypass without difficulty. Postoperative recovery was unremarkable apart from atrial fibrillation and bibasal atelectasis, managed medically. The patient was ready for discharge when her pathology report was released. Microscopic analysis showed clear resection margins but also strong staining for PAX8 and panCK suggestive of metastatic clear-cell RCC. Urgent consultations with urology, general surgery, and medical oncology were arranged. A computed tomography scan of the brain/neck/chest/abdomen/pelvis was performed and showed masses in the pancreatic tail and right adrenal gland concerning for recurrent disease.

## Discussion

LV metastases from RCC are extremely rare, particularly in the absence of vena cava extension. To our knowledge, there have been only several prior reports.^1^^,^^2^ Here, the patient had a distant history of malignancy and was deemed cleared of disease. The consensus was that the tumor was likely a myxoma. Cardiac myxomas account for approximately 50% of all primary intracardiac tumors, with ventricular involvement in up to 10%.^3^ Like this case, they are usually smooth, lobulated masses attached to the cardiac septum via a stalk. The recommended treatment is complete excision. In this case, the finding of RCC was surprising. Aburto and colleagues^1^ reported a late RCC recurrence manifesting as concomitant LV and pulmonary metastasis after 18 years. This attests to the slow growing nature of RCC and propensity to present years after complete resection. Given that RCC is highly resistant to chemotherapy, hormonal therapy, and radiation, surgery has been recommended for RCC with cardiac involvement.^1^ Improved overall and progression-free survival have been reported more recently with receptor tyrosine kinase inhibitors, including pazopanib.^4^ Nevertheless, this case highlights the caution should be exercised for any cardiac tumor that resembles a myxoma if there is a distant history of malignancy. It suggests that dedicated preoperative imaging for malignancy should be considered before multidisciplinary team-guided consideration for an operation.

The patient provided informed written consent for the utilization of de-identified data for this study; institutional review board approval was not required.

## Conflict of Interest Statement

The authors reported no conflicts of interest.

The *Journal* policy requires editors and reviewers to disclose conflicts of interest and to decline handling or reviewing manuscripts for which they may have a conflict of interest. The editors and reviewers of this article have no conflicts of interest.

## Supplementary Data

Video 1 TOE demonstrating left ventricular mass in diastolic contact with the anterior mitral valve leaflet. Video available at: https://www.jtcvs.org/article/S2666-2507(25)00271-8/fulltext

Video 2 Right coronary artery angiogram demonstrating contrast blush within mass. Video available at: https://www.jtcvs.org/article/S2666-2507(25)00271-8/fulltext
