# Supplementary figures and images for: More than meets the eye: Not a cardiac myxoma
Source: JTCVS Tech. 2025 Jul 3;33:158–9. doi: 10.1016/j.xjtc.2025.06.022 (PMC12529672; doi:10.1016/j.xjtc.2025.06.022)

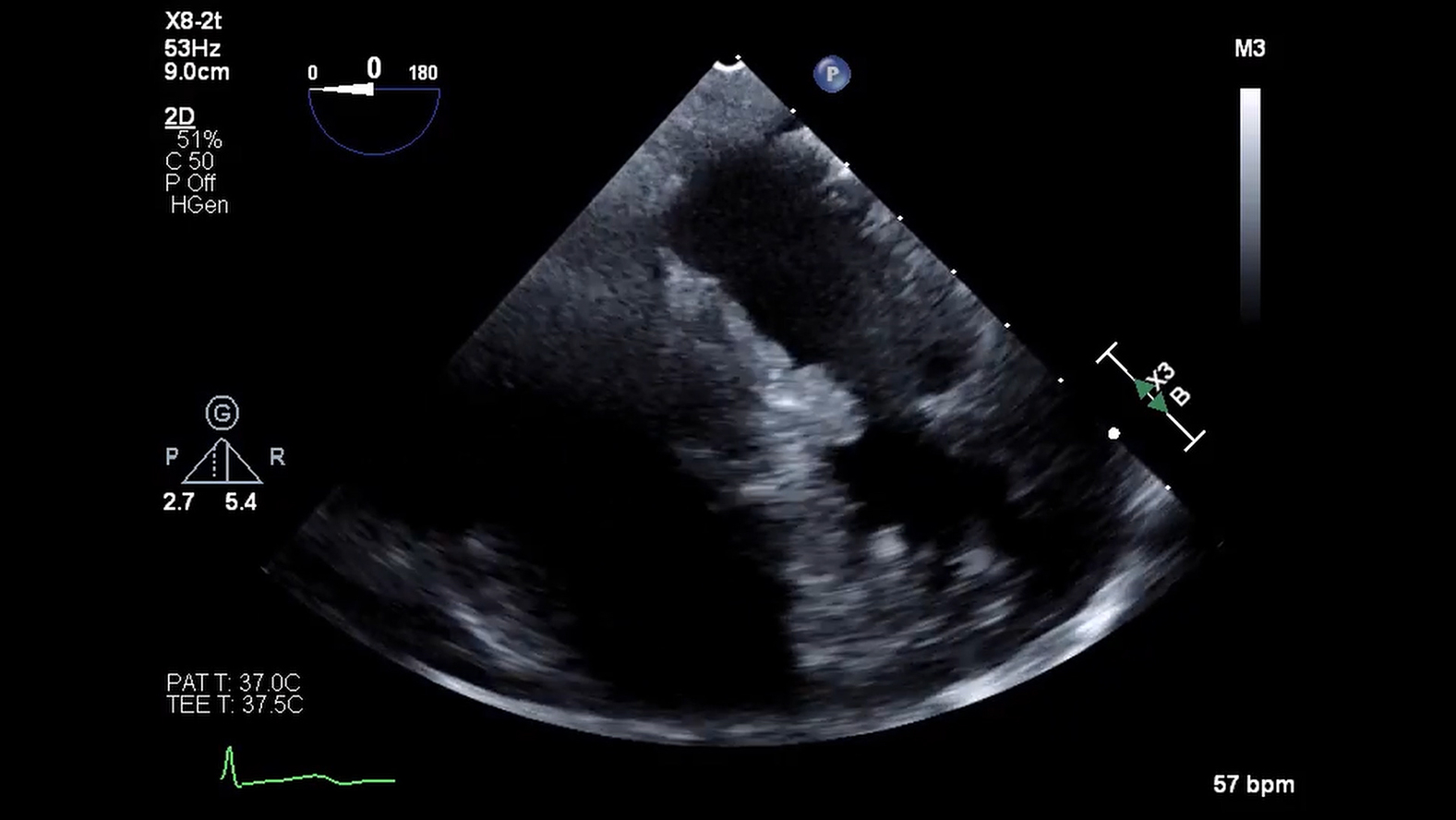

Supplement: Video 1 — TOE demonstrating left ventricular mass in diastolic contact with the anterior mitral valve leaflet. Video available at: https://www.jtcvs.org/article/S2666-2507(25)00271-8/fulltext [file fx2.jpg]

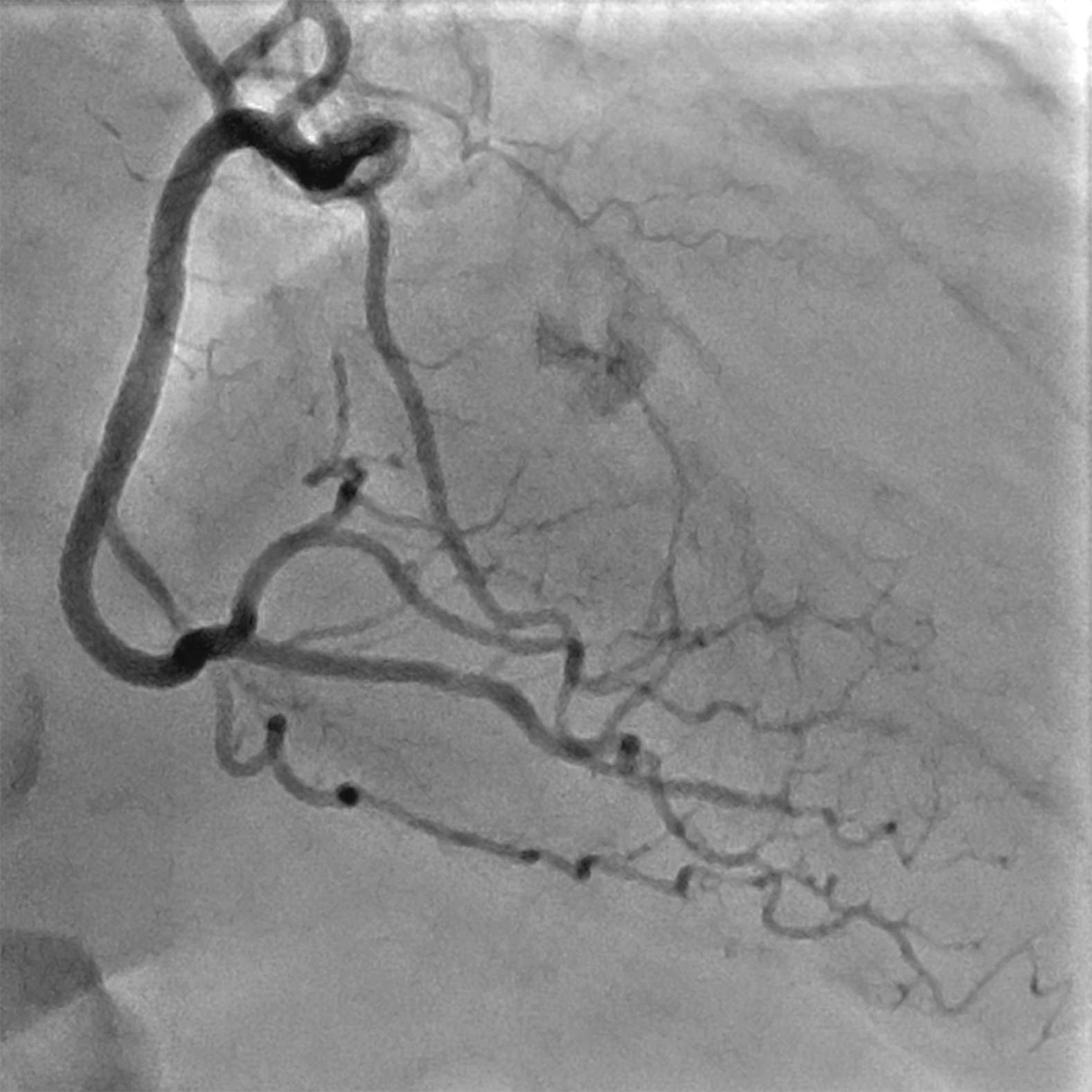

Supplement: Video 2 — Right coronary artery angiogram demonstrating contrast blush within mass. Video available at: https://www.jtcvs.org/article/S2666-2507(25)00271-8/fulltext [file fx3.jpg]
